# Mood symptoms contribute to working memory decrement in active-duty soldiers being treated for posttraumatic stress disorder

**DOI:** 10.1002/brb3.53

**Published:** 2012-07

**Authors:** Michael N Dretsch, Kenneth J Thiel, Jeremy R Athy, Clinton R Irvin, Bess Sirmon-Fjordbak, Anthony Salvatore

**Affiliations:** 1 U.S. Army Aeromedical Research LaboratoryFort Rucker, Alabama 36362-0577; 2 University of Texas-El PasoEl Paso, TX, USA

**Keywords:** Anxiety, depression, digit span, memory, military, neurocognitive

## Abstract

A significant proportion of military veterans of operations in Afghanistan and Iraq have been diagnosed with posttraumatic stress disorder (PTSD). Growing evidence suggests that neuropsychological deficits are a symptom of PTSD. The current study investigated neurocognitive functioning among soldiers diagnosed with PTSD. Specifically, active-duty soldiers with and without a diagnosis of PTSD were assessed for performance on tests of attention and working memory. In addition, factors such as combat experience, depression, anxiety, PTSD symptom severity, and alcohol consumption were explored as possible mediators of group differences in neurocognitive functioning. Twenty-three active-duty soldiers diagnosed with PTSD were matched with 23 healthy Soldier controls; all were administered the Attention Network Task (ANT), Backward Digit Span (BDS) task, Beck Depression Inventory, Beck Anxiety Inventory, PTSD Checklist—Military Version, Combat Exposure Scale, and Modified Drinking Behavior Questionnaire. Soldiers diagnosed with PTSD performed significantly worse on the working memory task (BDS) than healthy controls, and reported greater levels of PTSD symptoms, combat exposure, depression, and anxiety. However, after controlling for depression and anxiety symptoms, the relationship between PTSD and working memory was no longer present. The results indicate that PTSD is accompanied by deficits in working memory, which appear to be partially attributed to anxiety and depression symptoms.

## Introduction

The incidence of combat-related posttraumatic stress disorder (PTSD) continues to be a significant health concern for U.S. military soldiers currently serving under Operation Enduring Freedom (OEF) and Operation Iraqi Freedom (OIF). Surveys of military personnel returning from Afghanistan and Iraq suggest that anywhere between 8% and 17% of soldiers screen positive for PTSD symptoms (Hoge et al. 2004; Schneiderman et al. 2008; Smith et al. 2008).

The most common symptom of PTSD is persistent neuro-psychological trauma (e.g., flashbacks, high arousal and anxiety, intrusive memories); however, increasing evidence suggests that PTSD also produces both acute and chronic neurocognitive deficits (Horner and Hamner 2002; Yehuda et al. 2006; Marx et al. 2009; Vasterling et al. 2009; Lagarde et al. 2010; but see Crowell et al. 2002; Burris et al. 2008). For example, PTSD has been linked with poor performance on neuropsychological tests of working memory, attention, and executive functioning (Vasterling et al. 1998, 2002; Brandes et al. 2002; Veltmeyer et al. 2005; Samuelson et al. 2006; Johnsen et al. 2011). In contrast, some research has failed to identify impairments in visual attention and working memory associated with PTSD (Jenkins et al. 2000; Burriss et al. 2008).

Neurocognitive impairments associated with PTSD are commonly investigated. In particular, attention plays a crucial role in the modulation of affective responses. Evidence indicates that dysregulation of frontolimbic neural circuitry underlying emotional regulation is implicated in mood psychopathology (Siegle et al. 2007; Liao et al. 2012). Cognitive control of attention is one frontal mechanism involved in overriding a prepotent or conditioned responses. It is also implicated in trial-by-error learning and inhibiting irrelevant information during goal-directed behaviors. Considering the roles of working memory and emotional regulation, evidence indicates a bidirectional influence in the form of an inverse relationship due to disruption in the allocation of attentional resources (Dretsch and Tipples 2008; MacNamara et al. 2011).

Extensive evidence suggests that mild attentional impairments are associated with PTSD in both civilian and military populations (Uddo et al. 1993; Vasterling et al. 2002; Brewin et al. 2007; Leskin and White 2007; El Khoury-Malhame et al. 2011; Bomyea et al. 2012). However, attention is a complex function comprising multiple, casually independent networks with overlapping neural and neurobiological underpinnings (Posner and Petersen 1990; Fan et al. 2005). As such, the relationship between PTSD and attentional impairment is documented but needs further investigation to elucidate the differences between active-duty versus both civilians and veterans with PTSD.

The complexity of attentional processes can be captured using various laboratory tasks that rely on intact neural functioning of multiple networks and regions. The Attention Network Task (ANT; Fan et al. 2005) assesses the efficiency and independence of three primary attentional networks: alerting, orienting, and executive control (Callejas et al. 2004). Each of these measurable functions is correlated with specific neural networks and regions. Although the ANT is sensitive to attentional deficits associated with PTSD in civilians (Leskin and White 2007), it has yet to be attributed among a population of soldiers diagnosed with PTSD.

Research exploring the effects of PTSD upon the functioning of memory has yielded mixed results (Jenkins et al. 2000; Samuelson et al. 2006; Schweizer and Dalgleish 2011). Burriss and colleagues (2008) provide evidence that PTSD is associated with verbal memory impairments. Although memory impairments are in accordance with prior studies (Veltmeyer et al. 2005), the findings expand upon prior work by revealing that memory differences are mediated by depression scores, suggesting that deficits in learning and memory are mediated by frequency and intensity of depression.

In Burris et al. (2008), working memory function was within normal range among veterans diagnosed with PTSD. However, it is important to note that this domain was measured using the traditional forward digit span task. Compared to the forward digit span, the Backward Digit Span (BDS) task has been shown to load different cognitive processes in that it relies on internal manipulation of mnemonic representations and is not sensitive to the type of information being remembered (Conklin et al. 2000). Brain imaging suggests that the BDS task taxes specific regions of the dorsolateral prefrontal cortex more heavily than the forward digit span task (Hoshi et al. 2000). The BDS task is sensitive to working memory deficits from neurodegenerative disease, traumatic brain injury, and psychiatric illness (Conklin et al. 2000; Backman et al. 2001; Fork et al. 2005).

The current study investigated neuropsychological functioning associated with combat-related PTSD. We tested active-duty soldiers with PTSD versus healthy active-duty soldiers on specific components of attention using the ANT and working memory functioning as assessed with a BDS. Metrics of combat experience, depression, anxiety, PTSD, and alcohol consumption were explored as possible mediators of neuropsychological functioning.

## Materials and Methods

### Participants

Forty-six active-duty U.S. Army Soldiers (36 males) with prior combat experience voluntarily participated in this cross-sectional study. Participants were recruited via posters and clinician referral from a garrison PTSD treatment facility. All participants in the PTSD group (*n*= 23) had received, from a military health provider, an active diagnosis for PTSD (American Psychological Association 2000) as assessed via the Clinician Administered PTSD Scale (CAPS). The diagnosis had to be annotated in Military Health Service Electronic Medical Record or hard copy of medical record. Individuals were excluded if there existed a comorbid psychiatric diagnosis of a mood disorder, psychotic disorder (e.g., schizophrenia), or were currently being treated for substance dependency. The control group (*n*= 23) consisted of soldiers with no PTSD or any other mental health diagnosis, recruited from both patients and staff from different departments at the post Army Medical Center. Nine participants in the PTSD group (43%) reported having received a concussion or mild traumatic brain injury (mTBI) with a brief loss of consciousness (LOC) within the last five years. Individuals were excluded if they reported or had documented a moderate-to-severe TBI at any point in their medical history. All participants passed a screening for effort using the Test of Memory Malingering (TOMM) with a cut-off score of less than 45 correct.

All participants gave written informed consent and authorization for the health insurance portability and accountability act (HIPAA). The study was approved by the Institutional Review Board (IRB) at William Beaumont Army Medical Center, Fort Bliss, TX.

### Behavioral measures

#### Backward Digit Span (BDS) task

The BDS is based on the Weschler Digit Span test of working memory. Performance on the BDS involved presenting the participant with a random number series, four to eight digits paced at 1 sec per digit on the computer screen. Immediately afterwards, the participant was prompted to enter this series, but in reverse order. For example, if presented with the series “3–6-1–5,” the correct answer would be “5–1-6–3.” The task displayed six randomized trials per string length (4, 5, 6, 7, and 8 digits) for a total of 30 trials. Participants were provided with written instructions and verbally informed that they were not allowed to rehearse aloud, but instead, must rehearse in their head silently.

#### Attention Network Task (ANT; Fan et al. 2005)

The ANT allows for the identification of a specific deficit rather than suggesting the existence of an overall disturbance in performance. Subjects completed the task on a computer by clicking right and left mouse buttons to respond to visual stimuli. A row of five white lines with arrowheads pointing to either the left or to the right was visually presented. The target was a leftward or rightward pointing arrowhead at the center. The target was flanked on either side by two congruent (same direction) or incongruent arrows (opposite direction), or by neutral lines. The participant was to indicate as quickly as possible the direction of the central target by pressing the right or left mouse button. Our version of the task provided 240 randomized trials balanced for target type (congruent vs. incongruent) and cut positioning (no cue vs. double cue vs. center cue vs. directional cue).

### Questionnaires

#### Test of Memory Malingering (TOMM; Tombaugh 2003)

The TOMM was used to assess effort in all participants. The TOMM consists of two learning trials and a retention trial that uses pictures of common, everyday objects (e.g., chair, pencil). A cut-off score (<45 correct) for the first two learning trials was used to determine eligibility for participation in the study.

#### Beck Depression Inventory—2nd Edition (BDI-II; Beck and Steer 1984)

The BDI-II is a questionnaire designed to assess the severity of depression in adolescents and adults. The BDI-II consists of 21 items which participants rate on a 4-point scale ranging from 0 to 3 in terms of severity. The BDI-II has a high internal consistency (0.92) and test–retest reliability (0.93). A total score of 0–13 is considered minimal range, 14–19 is mild, 20–28 is moderate, and 29–63 is severe.

#### Beck Anxiety Inventory (BAI; Beck et al. 1988)

The BAI total score is the sum of the ratings for 21 symptoms. Each symptom is rated on a 4-point scale ranging from 0 to 3. It has a high internal consistency (0.93) and test–retest reliability (0.75). A total score of 0–21 is considered very low anxiety, 22–35 is moderate, and 36 or higher is severe.

#### PTSD Checklist—Military Version (PCL-M; Orsillo 2001)

The PCL-M is a 17-item self-report measure of the DSM-IV symptoms of PTSD. It has a test—retest reliability of 0.96, internal consistency (alpha coefficient) of 0.93 for B symptoms (i.e., re-experiencing), 0.92 for C symptoms (i.e., effortful avoidance), 0.92 for D symptoms (i.e., hyperarousal), and 0.97 for all 17 symptoms. A total score of 50 is considered to be PTSD positive in the military.

#### Combat Exposure Scale (CES; Keane et al. 1989)

The CES is a 7-item self-report measure that assesses wartime stressors experienced by combatants. Items are rated on a 5-point frequency, 5-point duration, 4-point frequency, or 4-point degree of loss scale. It has a test—retest reliability of 0.97 and internal consistency of 0.85. Cut-off scores for combat experience include light (0–8), light moderate (9–16), moderate (17–24), moderate heavy (25–32), and heavy (33–41).

#### Modified Drinking Behavior Questionnaire (DBQ; Cahalan et al. 1969)

The DBQ is a 10-item self-report measure of alcoholic drinking behavior consisting of separate items for rating average frequency of drinking occasions and average quantity of consumption per occasion over the past year. The task asks questions such as “On the average, how often do you consume alcoholic beverages of any kind?” Questions are rated on a 10-point scale. The DBQ has a test–retest reliability of 0.93 (Adair et al. 1996). Quantity was indicated by the number of drinks necessary to reach each of these states of inebriation. Each frequency/quantity pair was multiplied, summed, and divided by three to obtain a frequency/quantity index of alcohol use.

## Results

### Data reduction and analysis

Index scores for the alerting, orienting, and executive components of the ANT were calculated to assess the efficiency of individual attention networks by subtracting the mean response times between conditions; alerting (no cue–double cue), orienting (central cue–spatial cue), and executive attention (incongruent–congruent). Responses to the BDS test of working memory were scored for both individual trials for each string length and aggregated. For individual string lengths (values specifically pertaining to trials in which string lengths of 4, 5, 6, 7, or 8 digits were presented), proportions of correct responses were determined by scoring the correct responses for each trial and averaged. Each string length contained six trials. Aggregate scores for all phases were calculated by summing the proportion scores from all string lengths of the task.

### Descriptive variables

The data from 44 participants were included in the final analysis after removing three from the initial collection set due to incomplete data. Of the final participants, 21 (47.7%) had an active clinical diagnosis of PTSD while 23 (52.3%) served as controls. Among the final set, 34 participants were male and 10 were female. The female participants were disproportionately distributed between our diagnosis groups, with eight females (34.8%) included in the control group and two (9.5%) included in the PTSD group, χ^2^ (1, *n*= 44) = 3.99, *P* < 0.05, OR = 5.07. The number of participants reporting sleep difficulties in the control group (13%) was disproportionately lower than what was reported by the PTSD group (90%), χ^2^ (1, *n*= 44) = 26.33, *P* < 0.05, OR = 63.33. Eleven PTSD-diagnosed participants reported taking prescribed antidepressant medication (52.4%) as opposed to only two participants from the control group (8.7%), χ^2^ (1, *n*= 44) = 10.06, *P* < 0.05, OR = 11.55. The descriptive statistics for all other independent variables are presented in Table 1.

**Table 1:** Independent variables: descriptive statistics

Variable	Mean	SD	Min	Max
Age	35.43	8.51	20	58
Education	14.64	1.87	12	19
PCLM	40.61	18.95	17	74
CES	15.52	12.37	0	35
DBQ	27.98	12.41	10	53
BDI	13.32	10.92	0	37
BAI	12.80	13.12	0	44

PCLM, PTSD Checklist—Military; CES, Combat Exposure Scale; DBQ, Drinking Behavior Questionnaire; BDI, Beck Depression Inventory; BAI, Beck Anxiety Inventory.

### Self-report measures

A series of *t*-tests were conducted to explore observed group differences between PTSD-diagnosed participants and the control group with regard to independent variables (Table 2). Bonferroni adjustments to the alpha levels were made in order to correct for familywise error rate. As expected, PTSD-diagnosed participants reported experiencing more PTSD symptoms (M = 58.62, SD = 8.95) than did participants in the control group (M = 24.17, SD = 6.10), *t* (42) = 9.70, *P* < 0.0071. PTSD-diagnosed participants also reported higher levels of combat exposure (M = 24.76, SD = 9.40) than did participants in the control group (M = 7.09, SD = 7.92), *t* (42) = 6.77, *P* < 0.0071. PTSD-diagnosed participants reported higher levels of both depression, *t* (42) = 9.70, *P* < 0.0071, (M = 22.71, SD = 7.84) and anxiety (M = 24.43, SD = 9.53) than control participants (M = 4.74, SD = 4.00; M = 2.17, SD = 2.57, respectively), *t* (42) = 10.79, *P* < 0.0071. No group differences were observed regarding age, years of education, or alcohol consumption.

**Table 2:** Independent variables: comparisons of means between PTSD and control group (*t*-tests)

Variable	PTSD mean	Control mean	Difference
Age	37.57	33.48	4.09
Education	14.33	14.91	.58
PCLM	58.62	24.17	34.45*
CES	24.76	7.09	17.67*
DBQ	27.48	28.43	.95
BDI	22.71	4.74	17.97*
BAI	24.43	2.17	22.26*

* Significantly different at the 0.0071 (0.05) level using the Bonferonni method to control for familywise error rate.

### Behavioral performance

A multivariate analysis of variance (MANOVA) was conducted to explore group behavioral differences on BDS scores and alerting, orienting, and executive efficiency index scores of the ANT. Neither the assumption of homoscedasticity nor equal group variances were violated (*P* > 0.05). PTSD participants had significantly lower scores (M = 1.41, SD = 0.91) on the BDS task than did controls (M = 2.54, SD = 1.11), *F*(1, 42) = 13.35, *P*= 0.001, 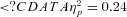. No other group differences were observed, *P* > 0.05 (Table 3).

**Table 3:** Dependent variables: comparisons of means between PTSD and control group

Variable	PTSD mean	Control mean
BDS mean*	1.41	2.54
Alerting (RT)	29.03	34.05
Orienting (RT)	23.90	35.00
Executive (RT)	−132.09	−128.89

* Significant effect for group observed when a MANOVA was conducted including all listed dependent variables and group as an independent factor.

BDS mean, Backward Digit Span aggregate scores; Alerting (RT), Attention Network Task (ANT) Alerting Index; Orienting (RT), ANT Orienting Index; Executive (RT), ANT Executive Attention Index.

For all ANT measures, negative values on reaction time (milliseconds) variables indicate the facilitation of faster responses to congruent versus incongruent trials.

### Working memory: aggregate scores

An analysis of covariance (ANCOVA) was conducted to explore the relationship between PTSD diagnosis and aggregate working memory (BDS) scores, while entering depression, anxiety, and combat exposure as covariates. A preliminary analysis evaluating the homogeneity-of-regression (slopes) assumption indicated that the relationship between the covariates and the dependent variable did not differ significantly as a function of the independent variable, *P* > 0.05. After adjusting for covariates, the relationship between PTSD and working memory observed in earlier tests was no longer significant, *F*(1, 42) = 1.77, *P*= 0.191, 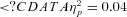. None of the three covariates were significantly related to working memory performance.

A series of mediation analyses were conducted to test if anxiety, depression, and combat exposure scores each served as total or partial mediators of the relationship between PTSD diagnosis and impaired working memory. Using the Freedman and Schatzkin (1992) test for partial mediation, neither depression, *t* (42) = 0.29, *P* > 0.05, anxiety, *t* (42) = 0.28, *P* > 0.05, nor combat exposure, *t* (42) = 0.31, *P* > 0.05, proved to meet the criteria for partial mediators of the relationship between PTSD diagnosis and working memory scores.

### Antidepressant use

The effects of antidepressant use upon working memory as measured by collapsed BDI scores were examined. Due to the low numbers of participants in the control group who reported taking some form of antidepressant medication at the time of data collection (*n*= 2), only data from participants diagnosed with PTSD were included for analysis. Among PTSD-diagnosed participants, there was no difference with regard to working memory as measured by collapsed BDS scores between those participants having reported using antidepressant medication (*n*= 11) at the time of data collection and those participants not reporting the use of antidepressant medication (*n*= 10), *t* (19) = 0.65, *P* > 0.05.

### Prior concussions and working memory

To assess possible additive effects of having a prior concussion, individuals in the PTSD group were placed into one of two subgroups based on reporting having a LOC from a head injury: (1) PTSD with a LOC (PTSD + LOC; *n*= 9) from a prior head injury and (2) PTSD with no LOC (PTSD – LOC; *n*= 12). BDS scores from both of these subgroups were compared against each other and with the control group. The results indicated that, although, both the PTSD + LOC group and PTSD – LOC group were significantly different from the control group, *t* (30) = 3.46, *P* < 0.05; *t* (33) = 2.41, *P* < 0.05; respectively; the difference between the PTSD + LOC and PTSD – LOC groups was not significant, *t* (19) =–1.16, *P* > 0.05. In as such, the results do not suggest there was a significant contribution of prior concussions on working memory function above and beyond that of PTSD alone. However, one of the limitations is the small sample size of the subgroups. Therefore, future studies should continue to explore the main effects and interactions of PTSD and concussion comorbidity on neurocognitive functioning.

### Working memory: individual phases

**Figure 1:**
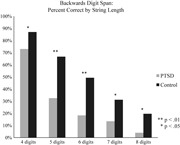
Group differences in working memory performance qualified as average percent correct by string length on the Backward Digit Span task. [Correction added after first online publication on 04 May 2012: The *P* values have been amended to **p < .01 and *p < .05].

A series of *t*-tests were conducted to determine if a relationship existed between PTSD and working memory on each of the individual string lengths (4, 5, 6, 7, and 8 digits) for reverse recall. The Holm procedure was used to correct for familywise error rate. For all string lengths, participants diagnosed with PTSD exhibited poorer working memory than participants in the control group (Fig. 1).

## Discussion

The main finding of this study is that active-duty soldiers diagnosed with combat-related PTSD demonstrate compromised working memory functioning as assessed by the BDS. Interestingly, controlling for depression, PTSD, and combat exposure eliminated the differences between the groups on the working memory task. In contrast, the soldiers did not differ from non-PTSD-diagnosed active-duty soldiers on measures of attention toward emotionally neutral visual stimuli.

A strong link between depression and compromised cognitive function has been established (Pio de Almeida et al. 2011; Doumas et al. 2012). Because there is a high prevalence of depression associated with PTSD (Hoge et al. 2004; Wright et al. 2011), there is reason to question if symptoms of depression mediated the decrements in working memory rather than psychopathological changes. The results of the current study did not provide support for depression, by itself, as full or partial mediator of working memory performance.

The present findings are somewhat at odds with a report by Burriss and colleagues (2008) who failed to find working memory impairments in veterans diagnosed with PTSD. In contrast to previously published studies, our findings did not reveal a relationship between PTSD and cognitive control of attention (Leskin et al. 2007). Although working memory is tested in both the present study and Burriss et al. study, each used differing memory indices and methodological differences must be accounted for when considering disparate study findings.

Participants in the Burriss et al. study consisted of veterans with PTSD recruited from patients visiting primary care clinics at a VA Medical Center. In contrast, the current study used active-duty soldiers being treated for PTSD at a Behavioral Health Department and/or a PTSD treatment facility. Typically, with Veteran Studies, the mean age is higher than that of our participants. For example, the mean age for the PTSD group reported by Burriss et al. is 52.1 years compared to 35.4 years in the current study. This might suggest that our younger sample of participants have compromised neurocognitive function with characteristics different from older populations derived from veterans and civilians. Hence, such variability between population neurocognitive profiles might be attributed to temporally related pathophysiological changes associated with either treatment or chronic hypothalamic pituitary axis (HPA) activation. Alternatively, test administration procedures might have resulted in increased variability in performance. For example, Burriss et al. administered the behavioral testing and self-report questionnaires on two separate sessions separated by one week, therefore, not taking into consideration changes in mood state.

Interestingly, a significant number of participants in the PTSD group in the current study were being treated with psychotropic medication (52.4%). Although the PTSD group was being treated pharmacologically, they still reported significant anxiety, depression, and PTSD symptoms compared to the control group. The post hoc analysis revealed that compared to nonmedicated participants, individuals on psycho-tropics had significantly higher depression scores. These findings might suggest antidepressants for treating PTSD-related affective symptoms may lack efficacy overall.

### Limitations

Practical considerations in the execution of this research resulted in limitations in the sample size. Although the number of participants for which data were obtained is large enough to ensure reliable and interpretable analyses, the relatively small number of participants in each group limited the possibility of observing factors and interactions with small effect sizes. The sample size was, however, determined by an a priori power analysis large enough to detect the expected and observed large effect sizes associated with the effects of PTSD upon working cognitive performance. Furthermore, similar sample sizes have been used in prior PTSD studies (Neylan et al. 2004; Yehuda et al. 2005).

Although the difference in working memory performance was no longer present when symptoms of depression and PTSD, and combat exposure were controlled for, tests of full and partial mediation of these variables to PTSD diagnosis produced inconclusive results. The limitations in sample size reduced the ability to determine the exact nature of the interrelationships between PTSD and other independent variables concerning their independent and combined effects upon cognitive performance. It was expected that PTSD diagnosis would contribute to cognitive deficits even after controlling for the effects of the depression and anxiety associated with PTSD. Specifically, it was expected that both anxiety and depression would serve as partial mediators of the relationship between PTSD and cognitive functioning, with PTSD contributing to increased levels of depression and anxiety that then contributed to increased deficits in cognitive functioning while independent variance from each variable still contributed to additional increases in cognitive deficits. The small sample size, in conjunction with high observed multicollinearity between independent variables, may have limited this study's power with regard to uncovering these partial mediation relationships.

Other factors associated with PTSD, such as reduced sleep quantity and quality, are known to influence neurocognitive functioning. Toblin and colleagues (2012) recently reported that almost 33% of soldiers experience sleep problems after deployment. Sleep problems have also been shown to be linked with changes in depression and PTSD symptoms in soldiers after deployment (Wright et al. 2011). Not unexpectedly, in the current study, 90% of the PTSD reported having sleep problems compared to only 13% in the control group. Equally relevant is the plethora of evidence that sleep quantity and quality can impact cognition (Schmidt et al. 2011). Due the small sample size and type of data collected, the current study is limited by power constraints to investigate the contribution of sleep to working memory performance. Future PTSD should strongly consider study designs that allow for exploring the interactions between sleep, PTSD, and neurocognitive functioning.

## Conclusion

The findings in the current study suggest that PTSD is associated with compromised levels of working memory functioning. However, as implied by the current study's findings, these cognitive impairments are likely to be attributed to psychological factors such as symptoms of depression and anxiety associated with overall combat exposure.
